# Long-term caloric restriction activates the myocardial SIRT1/AMPK/PGC-1α pathway in C57BL/6J male mice

**DOI:** 10.29219/fnr.v64.3668

**Published:** 2020-01-29

**Authors:** Lina Ma, Rong Wang, Hongjuan Wang, Yaxin Zhang, Zhiwei Zhao

**Affiliations:** 1 Department of Geriatrics, Xuanwu Hospital Capital Medical University, National Clinical Research Center for Geriatric Diseases, Beijing, China; 2 Central Laboratory, Xuanwu Hospital Capital Medical University, Key Laboratory for Neurodegenerative Disease of Ministry of Education, Center of Alzheimer's Disease, Beijing Institute for Brain Disorders, Beijing, China; 3 Department of Biochemistry and Molecular Biology, Yanjing Medical College, Capital Medical University, Beijing, China

**Keywords:** caloric restriction, signaling pathway, SIRT1, AMPK, mTOR

## Abstract

**Background:**

Caloric restriction (CR) can help in improving heart function. There is as yet no consensus on the mechanism of the effect of CR. Silent mating-type information regulation 1 (SIRT1), adenosine monophosphate-activated protein kinase (AMPK), and mTOR are key players in metabolic stress management. We aimed to explore the effect of CR on the myocardial SIRT1/AMPK/mTOR pathway in mice.

**Methods:**

Thirty-six 6-week-old male C57BL/6J mice were randomly divided into three groups: normal control group (NC group, *n* = 12), high-energy group (HE group, *n* = 12) and CR group (*n* = 12) according to different diets. After 11 months, western blot was used to examine proteins such as p-AMPK, peroxisome proliferator-activated receptor gamma coactivator 1-alpha (PGC-1α), SIRT1, and p-mTOR, whereas real-time PCR was used to examine the expression of AMPK, PGC-1α, and SIRT1 transcripts.

**Results:**

Compared to the HE group, the CR group displayed increased expression of myocardial p-AMPK protein, SIRT1 protein and mRNA, and PGC-1a mRNA. However, no difference was observed in the expression of p-mTOR protein and mTOR mRNA in the myocardium among the three groups.

**Conclusions:**

CR improves the SIRT1/AMPK/PGC-1α pathway in mice myocardium with no effect on the mTOR pathway.

## Popular scientific summary

- CR activates the SIRT1/AMPK pathway in mice myocardium.
- CR activates the myocardial PGC-1α pathway.
- CR has no effect on the myocardial mTOR pathway.

Caloric restriction (CR) has been shown to prolong the lifespan of rodents in addition to lowering body temperature, blood sugar, insulin levels, glucagon levels, fat, and body weight, along with an increase in insulin sensitivity and organ volume (except that of the brain) (1, 2). Triglycerides, fasting blood glucose, and insulin levels have also been observed to decrease upon subjecting rhesus monkeys to CR (3). Short-term CR can reduce body weight and significantly improve cardiovascular risk factors such as body mass index, waist circumference, hip circumference, waist-to-hip ratio, total cholesterol, and serum triglycerides in humans (4). For non-obese people, CR can also significantly reduce serum cholesterol, triglycerides, fasting blood glucose, fasting insulin levels, as well as systolic and diastolic blood pressure (5). CR or CR combined with exercise can reduce body weight, thereby reducing vascular risk factors such as blood lipids and blood pressure in healthy obese people (6). Short-term CR can improve insulin sensitivity and reduce the risk of cardiovascular diseases, indicating its potential benefit for human health. Silent mating-type information regulation 1 (SIRT1) agonist-resveratrol has protective effects on cardiovascular function, lipid lowering, increased glucose tolerance, and insulin sensitivity (7). These effects mimic the cardiovascular protective role of CR by reducing platelet aggregation, cholesterol, and triglycerides, and promoting vascular relaxation, anti-atherosclerosis activity, resistance toward oxidative stress, protection against myocardial infarction, heart failure, and other diseases. CR can alleviate diastolic dysfunction and decreased myocardial activity in mice in addition to reducing the expression of aging-related genes in the heart by 90% (8). Thus, CR has the potential to reduce the incidence of cardiovascular diseases, associated complications, and mortality by regulating the systemic cardiovascular risk factors.

We have previously reported that CR improves the spatial learning ability in mice, mainly through the SIRT1/adenosine monophosphate-activated protein kinase (AMPK)/mTOR signaling pathway (9). CR is capable of improving the cardiovascular risk factors such as glycolipid metabolism and insulin resistance, but the mechanism of cardiovascular protection remains unclear. SIRT1 is known to reduce inflammation, possess anti-atherosclerosis activity, inhibit cardiomyocyte apoptosis and telomere shortening, resist myocardial oxidative stress damage and myocardial remodeling, maintain myocardial energy balance, and promote autophagy (7). AMPK can inhibit cell proliferation by affecting the cell cycle and metabolism when myocardial and vascular smooth muscle cells are stimulated by ischemia/hypoxia or cardiovascular active substances, playing an important role in the cardiovascular system (10, 11). However, it remains unelucidated whether or not the cardiovascular protection of CR is mediated through the SIRT1/AMPK/mTOR pathway Therefore, we aimed to observe the effects of different energy diets on the myocardial SIRT1/AMPK pathway and provide theoretical basis for the cardiovascular protective effects of CR.

## Methods

### Experimental animals

Thirty-six 6-week-old male C57/BL mice from the Academy of Military Medical Sciences (Beijing, China) were fed *ad libitum* for 1 week before the experiment began. All animal study protocols were approved by the Institutional Animal Care and Ethics Committee of Xuan Wu Hospital, Capital Medical University in Beijing, China.

Thirty-six 6-week-old male C57BL/6J mice were randomly divided into three groups: normal control group (NC group, *n* = 12), high-energy group (HE group, *n* = 12) and CR group (*n* = 12) according to different diets. The food composition of NC diet, HE diet, and CR diet is shown in Table 1, and the NC:HE:CR caloric ratio was 1:1.3:0.7. Food consumption data were collected manually daily to ensure each mouse had a consistent food intake. After 11 months, both the body weight and blood glucose were lower in the CR group than in the NC group and the HE group (Table 1).

**Table 1:** The food composition, body weight, and blood glucose of the three groups

Item	Food composition	Energy of diet (kcal/g)	Body weight (g)	Blood glucose (mmol/L)
Normal control (NC) group	19.1% protein, 4% fat, 59% carbohydrate	3.484	26.73 ± 5.49	4.52 ± 0.72
Caloric restriction (CR) group	19.1% protein, 2.5% fat, 37.2% carbohydrate	2.479	22.01 ± 2.31	3.99 ± 0.60
High-energy (HE) group	19.1% protein, 21.5% fat, 47.2% carbohydrate	4.589	35.04 ± 7.65	5.83 ± 1.04

### Western blot analysis

After 11 months, animals were sacrificed and hippocampus tissues were collected for western blotting and real-time polymerase chain reaction (PCR) analysis. The following primary antibodies were kept at 4°C overnight: rabbit anti- SIRT1 (1 : 2,000, Abcam, Cambridge, UK), rabbit anti-p-mTOR (1 : 2,000, Cell Signaling, Beverley, MA, USA), rabbit anti-p-AMPK (1 : 2000, Cell Signaling, Beverley, MA, USA), and rabbit anti-peroxisome proliferator-activated receptor gamma coactivator 1-alpha (PGC-1α) (1 : 1000, Cell Signaling, Beverley, MA, USA). After rinsing with TBS-T, the membranes were incubated with a goat anti-rabbit horseradish peroxidase-(HRP-) conjugated immunoglobulin (Ig)G (H + L) secondary antibody (1 : 20,000, Beijing TDY Biotech Co. Ltd.) for 40 min at room temperature.

### Real-time PCR

The hippocampus tissues were collected for real-time PCR analysis. Total RNA was extracted using an RNA Extraction Kit (CWBio Co., Ltd., Beijing, China) and reverse-transcribed using an ExScript RT reagent kit (CWBio Co., Ltd.). Real-time PCR was then performed using an ABI 7500 system (Applied Biosystems, Foster City, CA, USA) and UltraSYBR Mixture (CWBio Co., Ltd.). The specific primers were SIRT1, forward, 5′-TATGACGCTGTGGCAGATTGTTATT-3′; reverse, 5′-CCACCGCAAGGCGAGCAT-3′; AMPK, forward, 5′- AGCCAAATCAGGGACTGCTACT -3′; reverse, 5′- AGGGAGGTGACAGATGAGGTAAG -3′; mTOR, forward, 5′- TTCAATCCATAGCCCCGTCT -3′; reverse, 5′- CAAAGAGCTGCATCACTCGT -3′; PGC-1α, forward, 5′- GCUC UUGAGAAUGGAUAUATT -3′; reverse, 5′- UAUA UCCAUUCUCAAGAGCTT -3′; actin, forward, 5′-GCCTTCCTTCTTGGGTAT-3′; reverse, 5′-GGCATAGAGGTCTTTACGG-3′. The relative expression of amplified RNA samples was calculated using the 2-^ΔΔCT^ method.

### Statistical analysis

Data were analyzed using the SPSS 11.5 software (Chicago, IL, USA) and plotted as mean ± standard deviation (SD). Comparisons among the groups of animals were made with one-way analysis of variance (ANOVA) with a *post hoc* Tukey’s test. Results were considered to be significantly different at *P* < 0.05.

## Results

**Fig. 1:**
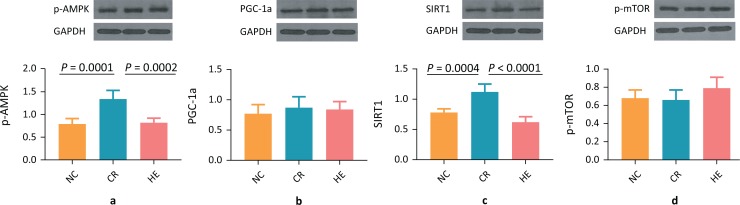
The translational effect of caloric restriction on the myocardial SIRT1/AMPK/mTOR pathway. (a) p-AMPK, (b) PGC-1α, (c) SIRT1, and (d) p-mTOR.

**Fig 2:**
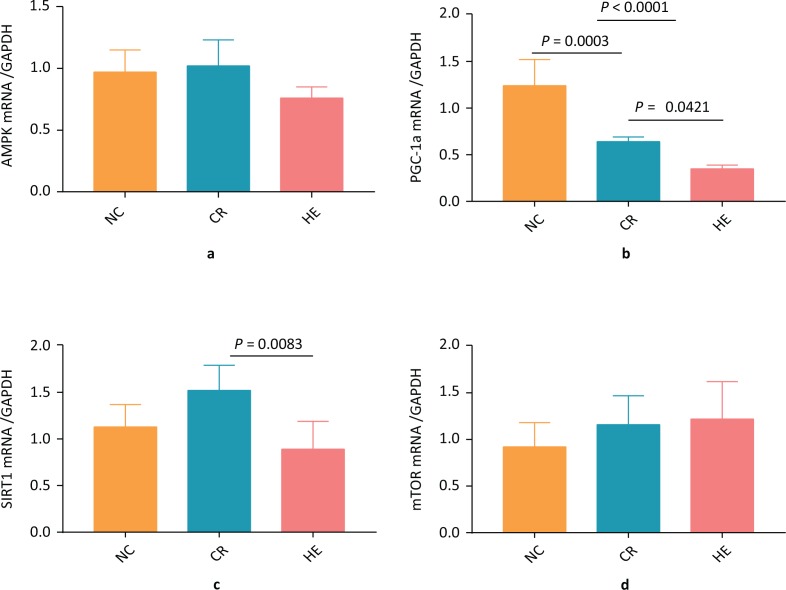
The transcriptional effect of caloric restriction on the myocardial SIRT1/AMPK/mTOR pathway. (a) AMPK, (b) PGC-1α, (c) SIRT1, and (d) mTOR.

To determine the association of CR with activation of the SIRT1/AMPK/mTOR pathway, a group of C57BL/6J mice was subjected to a CR diet along with an HE diet as well as the NC group of mice. After 11 months, the myocardial SIRT1 expression levels were analyzed using western blotting. The results revealed that both protein and transcript levels of myocardial SIRT1 were elevated in the CR group compared to the HE group (Figs. 1c and 2c), suggesting that CR activates SIRT1 to exert its cardiovascular protective effect. Compared with both the NC group and HE group, the protein levels of myocardial p-AMPK were increased in the CR group (Fig. 1a), but the difference in transcript levels was statistically insignificant. Furthermore, no significant difference was observed in myocardial PGC-1α protein levels between the three groups (Fig. 1b). However, the PGC-1α mRNA expression was significantly augmented (Fig. 2b). Nevertheless, no significant difference was observed in myocardial p-mTOR protein and transcript expression between the CR, NC, and HE groups (Figs. 1d and 2d).

## Discussion

Compared with the NC group and the HE group, the protein expression of p-AMPK and SIRT1 was higher in the CR group. The transcript levels of SIRT1 and PGC-1α showed an increase but there was no significant difference in the protein and mRNA levels of p-mTOR between the three groups, suggesting that the role of CR in cardiovascular function may be mainly mediated through the SIRT1/AMPK pathway. Studies have established that CR can improve insulin sensitivity, and reduce cardiovascular risk by controlling cardiovascular risk factors (12); however, its specific biological basis remains uncertain. In mammals, although different nutrient contents are perceived by different signaling pathways, CR is controlled by not a single but multiple signaling pathways. We have confirmed that CR in the early stage exerts neuroprotection and is associated with signaling pathways such as insulin, SIRT1, AMPK, and mTOR signaling pathways.

### CR activates the myocardial SIRT1 pathway

Our results showed that the expression of SIRT1 protein and mRNA in the CR group is elevated as compared to that in the HE group. This finding thus suggests that CR can activate SIRT1 for exerting its cardiovascular protection. The *SIRT1* gene plays a critical role in the growth of animals. The SIRT1-/- animals demonstrate reduced environmental adaptability and higher postnatal mortality, considering the possible regulation of energy metabolism due to the lack of SIRT1 deacetylase (13). In transgenic mice, moderate expression of SIRT1 improves the glucose tolerance and insulin sensitivity in addition to resistance toward diabetes (14). However, overexpression of SIRT1 enhances glucose tolerance as well as the metabolic rate (15). Furthermore, administration of the SIRT1 activator in mice fed with a high-fat diet results in development of resistance toward weight gain and insulin resistance caused by a high-fat diet (16, 17).

SIRT1 is a well-established anti-atherosclerotic factor *in vivo* and its anti-atherosclerotic effect is mediated through endothelial nitric oxide synthase (eNOS) expression, inhibiting endothelial cell apoptosis, improving endothelial function, and inhibiting E-selectin expression. In addition, reducing leukocyte adhesion to endothelial cells, promoting endothelium-dependent vasodilation, and increasing nitric oxide (NO) bioavailability are other modes by which SIRT1 exerts its anti-atherosclerotic effects. Studies have shown that while SIRT1 inhibitors decrease eNOS expression, an endothelial cells-specific over-expression of SIRT1 results in up-regulated expression of eNOS and improved vascular endothelial cell-dependent aortic diastolic function (18, 19). Furthermore, a reduction in aortic SIRT1 expression is consistent in individuals with a high-fat diet and is a risk factor for atherosclerosis (20). Reportedly, 3–12 months of CR induces the production of NO, formation of cGMP, increase in oxygen consumption, and ATP mitochondrial production in adult mouse endothelial cells, accompanied by enhanced expression of SIRT1 (21). CR significantly improves the expansion of NO-mediated resistance vessels in the skeletal muscles of aged F334 rats, suggesting that CR may improve the NO bioavailability as well as endothelial function (22, 23) and the mechanism may be associated with the regulation of eNOS expression by activating SIRT1. SIRT1 and eNOS co-localize in vascular endothelial cells where SIRT1 regulates the activity and endothelial secretion of NO by acetylating eNOS (19). Similarly, it has been found that overexpression of SIRT1 or the SIRT1 activator in the CR mice can induce the acetylation of eNOS and increase its expression in vascular endothelial cells (24). In the endothelial dysfunction animal model, the SIRT1 agonist resveratrol activates eNOS, enhances endothelial function, prevents elevated blood pressure, and restores vascular eNOS activity (19). For elderly CR individuals, it is unclear whether SIRT1 activators can increase NO bioavailability and improve vascular endothelial cell function.

Up-regulation of SIRT1 has a cardio-protective effect while inhibiting its activity in cardiomyocytes results in an increase in the rate of apoptosis and myocardial gene-related hypertrophy (25). Cardiomyocytes with high expression of SIRT1 are resistant to cardiomyocyte shrinkage and death (26). In primary cultured mouse ventricular myocytes, increased expression of SIRT1 results in resistance to myocardial apoptosis and significantly increases myocardial cells (25). The underlying mechanism for this phenomenon is postulated to be reduction of p53 activity upon deacetylation by SIRT1, thus reducing the apoptosis in cardiomyocytes. High expression of SIRT1 can also inhibit caspase-3 activity, thereby improving the ability of cardiomyocytes to resist apoptosis or aging, and protect the myocardium (26). Similar results are observed in case of cardiomyocytes subjected to oxidative stress (27).

### CR improves the myocardial AMPK pathway

The protein expression of p-AMPK in the CR group was elevated but the difference in the transcript levels was not statistically significant. Moreover, there was no significant difference in the PGC-1α protein level between the three groups, but its transcript level was significantly increased. PGC-1α is a substrate for SIRT1 deacetylation. Deacetylation of PGC-1α can increase its transcriptional activity.

It is found that knocking out the AMPK subunit leads to a significant reduction in PGC-1α phosphorylation and deacetylation (28). The resultant decrease in transcriptional expression and activity of PGC-1α is a hallmark of cardiac dysfunction and further results in impaired energy metabolism, leading to the development of systolic heart failure. In isolated hearts of AMPKα2 knockout mice, myocardial ischemia leads to a decline in glucose uptake compared to wild-type mice (29). Appropriate short-term CR improves endothelial function and lowers blood pressure in obese patients by activating the AMPK–PI3K–Akt–eNOS signaling pathway (30). Cardiac hypertrophy and AMPK activation during heart failure make the heart more dependent on glycolysis increasing ATP production. Upon AMPK mutation or gene knockout in myocardial cells, there is accumulation of glycogen which damages the myocardium (31). AMPK promotes the oxidation of free fatty acid by endothelial cells, antagonizes endothelial cell lipotoxicity caused by FFA, lowers cholesterol levels, and exerts protective effects on cardiovascular function (32). AMPK activation also inhibits caspase-3 activity in vascular endothelial cells induced by high glucose and reduces apoptosis (33). In addition, AMPK is an important regulator of autophagy in ischemic cardiomyocytes. Ischemia triggers AMPK-dependent autophagy, which has cardioprotective effects (34). Metformin activates AMPK and enhances eNOS activity to improve myocardial injury in diabetic and non-diabetic mice after ischemia-reperfusion (35), enhancing left ventricular function and viability, possibly by inhibiting the activity of complex I in the mitochondrial respiratory chain. This leads to high AMP/ATP levels, which in turn regulates AMPK activity (36). Although AMPK is important in the process of energy metabolism, it can simultaneously stimulate glucose and fatty acid uptake and utilization and has dual efficacy in metabolic failure of heart or cardiac hypertrophy.

SIRT1 is vital for AMPK activity while both share a common activator and target molecule (37). Studies have shown that activation of SIRT1 promotes transcription of energy metabolism-related genes and prevents ATP reduction (38). At the same time, activation of AMPK-induced FoxO/DAF-16, nuclear factor-erythroid 2-related factor 2/SKN-1, and SIRT1 signaling pathways enhances cell’s ability to resist stress (39). AMPK activates SIRT1 protein under various conditions of stress such as energy limitation and exercise (28). On one hand, CR increases the AMP/ATP ratio and AMPK activity; on the other hand, it raises NAD+ levels and activates SIRT1. Activated SIRT1 and AMPK act together to activate PGC-1α through phosphorylation and deacetylation respectively, causing mitochondrial biogenesis and fatty acid oxidation. Thus, AMPK and SIRT1 control each other’s activities, forming a regulatory loop (40).

### CR has no effect on myocardial mTOR pathway

Our study revealed no significant difference in myocardial p-mTOR protein expression between the three groups, suggesting that AMPK may not function by activating autophagy in the myocardium of CR mice. Autophagy plays an important role in the pathogenesis of cardiovascular diseases as effective autophagy clearance and increased stress resistance can prevent cardiovascular diseases (41). SIRT1 can directly activate autophagy. It has been found that SIRT1 deacetylated FoxO1 induces autophagy by increasing the expression of the Ras-related protein Rab7 in cardiomyocytes which stimulates lysosomal damage of phagosomes (42). SIRT1 can also induce autophagy by directly deacetylating autophagy proteins such as Atg5, Atg7, and Atg8 (43), which enhances phagosome formation. In liver kinase B1-deficient myocardium, the activation of the AMPKα2 subunit is restricted resulting in increased activity of the mTOR signaling pathway; insufficient energy; increased expression of vascular endothelial growth factor; and impaired cardiac function (44). Akt2 knockout significantly increases the effect of CR on mTOR and unc-51 like kinase 1, suggesting that Akt2 knockdown may protect against pathological changes caused by autophagy to balance the heart and long-term restriction of caloric intake (45). We speculate that the protective effect of CR on myocardium activates SIRT1/AMPK, but the downstream signaling is not mediated through the mTOR signaling pathway.

## Conclusion

Subjecting young C57BL/6J male mice to a CR diet, an HE diet, and normal feed (NC group) for 11 months results in enhanced expression of p-AMPK and SIRT1 at translational levels in the CR group as compared to the NC and HE groups. The expression of SIRT1 and PGC-1α transcripts was augmented; however, there was no significant difference in the protein and transcript levels of p-mTOR between three groups. These findings suggest that CR may play a cardiovascular protective role mainly through the SIRT1/AMPK/PGC-1α pathway with no involvement of the mTOR pathway. The SIRT1/AMPK/PGC-1α pathway is a key regulator of cardiac metabolism. Mitochondrial biosynthesis and fatty acid oxidation are induced downstream of PGC-1α, but its specific role needs further exploration.

## Conflict of interest and funding

The authors report no relationships that could be construed as a conflict of interest. This work was supported by the following grants: Beijing Natural Science Foundation (7202059, 7174310), Beijing Municipal Administration of Hospitals Incubating Program (PX2020036), and Nation Natural Science Foundation of China (81600927).
